# Passive Proprioceptive Training Alters the Sensitivity of Muscle Spindles to Imposed Movements

**DOI:** 10.1523/ENEURO.0249-21.2021

**Published:** 2022-01-28

**Authors:** Rochelle Ackerley, Léonard Samain-Aupic, Edith Ribot-Ciscar

**Affiliations:** Aix Marseille Univ, CNRS, LNC (Laboratoire de Neurosciences Cognitives – UMR 7291), 13331 Marseille CEDEX 03, France

**Keywords:** gamma fusimotor system, microneurography, muscle afferent, proprioception, training

## Abstract

Humans rely on precise proprioceptive feedback from our muscles, which is important in both the acquisition and execution of movements, to perform daily activities. Somatosensory input from the body shapes motor learning through central processes, as demonstrated for tasks using the arm, under active (self-generated) and passive conditions. Presently, we investigated whether passive movement training of the ankle increased proprioceptive acuity (psychophysical experiment) and whether it changed the peripheral proprioceptive afferent signal (microneurography experiment). In the psychophysical experiment, the ankle of 32 healthy human participants was moved passively using pairs of ramp-and-hold movements in different directions. In a pretraining test, participants made judgements about the movement direction in a two-alternative forced choice paradigm. Participants then underwent passive movement training, but only half were cued for learning, where a reference position was signaled by a sound and the participant had to learn to recognize this position; they then completed a post-training test. In a paradigm using the same setup, nine healthy participants underwent microneurography recordings of Ia muscle afferents from the peroneal nerve, where all were cued during training. In the psychophysical experiment, proprioceptive acuity improved with training only in the cued group. In the microneurography experiment, we found that muscle afferent firing was modulated, via an increase in the dynamic index, after training. We suggest that changes in muscle afferent input from the periphery can contribute to and support central perceptual and motor learning, as shown under passive conditions using ankle movements, which may be exploited for movement rehabilitation.

## Significance Statement

When you train your body to execute a movement, from walking to a complex sequence of movements required in sport, you develop your sense of movement (i.e., proprioception) to provide accurate feedback for precise control. This can be acquired actively or passively, through the modification of central processes. Here, we show that training induced through passive ankle movements can increase perceptual proprioceptive acuity and that these changes are, at least in part, signaled in the periphery directly from muscle afferent firing. This has potential implications in movement rehabilitation, especially concerning the leg and foot, and for those who have difficulties in self-generating movements.

## Introduction

Proprioceptive feedback is important in all stages of movement, including in its preparation, acquisition, and execution, as well as assisting in both habitual and high-skill performance. Walking is an example of how we routinely use our legs, where precise control of foot angle is crucial to decrease the risk of catching the ground during the swing phase of gait (Chen et al., 1991), which risks ankle injury (Waddington and Adams, 2003). Better lower limb proprioceptive acuity contributes to the acquisition of skilled walking tasks (Qaiser et al., 2016), and higher ankle proprioceptive sensitivity is a good predictor of achievement in elite sports, such as dancing (Han et al., 2015).

Earlier work investigated whether proprioception could be improved by training (for review, see Ashton-Miller et al., 2001), and, recently, it has been demonstrated that somatosensory processes are modified during the acquisition of a new motor skill, both when learning involves physical practice (Ostry et al., 2010; Wong et al., 2012; Vahdat et al., 2014; Haith et al., 2016) and through movement observation (Bernardi et al., 2013). Somatosensory input plays a fundamental role in motor learning, which can occur under both active and passive conditions (Darainy et al., 2013; Bernardi et al., 2015; Dimitriou, 2016). For example, this has been demonstrated using passive movements that were reinforced close to target location, where learning target position in a reaching task was shown to be comparable to that using active movements, highlighting the specific importance of afferent sensory information in motor learning (Bernardi et al., 2015). Sensorimotor plasticity following training has also been shown to increase with proprioceptive acuity (Ostry et al., 2010; Wong et al., 2012; Darainy et al., 2013; Bernardi et al., 2015).

Studies have shown that the differential processing of proprioceptive inputs following learning results in changes in the connectivity of central somatosensory networks. This has been demonstrated using resting-state functional magnetic resonance imaging (Vahdat et al., 2014) and electroencephalography, where the potential amplitude correlates with learning (Nasir et al., 2013). However, somatosensory contributions to human motor learning may not be solely central, but also involve the modulation of the peripheral proprioceptive sensory feedback itself, as recently demonstrated in a visuomotor adaptation task (Dimitriou, 2016).

Muscle spindles are mechanoreceptors in our muscles that contribute to the sense of body position and movement, but have the particularity of being influenced by CNS, which can change their sensitivity, via the gamma fusimotor drive (Matthews, 1972; Ribot-Ciscar and Ackerley, 2021). In humans, it is believed that this descending control of muscle spindle sensitivity works to prevent the slackening of muscle receptors when the parent muscle is shortened (for review, see Gandevia, 1996; Prochazka, 1996; Vallbo et al., 1979 ). However, the gamma fusimotor system is more complex, as demonstrated in studies in both active and passive situations, where muscle spindle sensitivity may be changed through this descending modulation, making the muscle receptors adapt to the behavioral context (Hospod et al., 2007; Ribot-Ciscar et al., 2009; Dimitriou, 2016; Ackerley et al., 2017) or task goal (Papaioannou and Dimitriou, 2021).

The influence of the descending gamma drive has been shown in studies varying cognitive factors. When attention is directed to imposed movements in order recognize a trajectory, the gamma drive increases to provide the brain with movement information that is more accurate (Hospod et al., 2007). Further, when a participant is asked to either focus on the amplitude or velocity of an imposed movement, to specifically trigger a static or dynamic fusimotor drive, respectively, muscle afferent feedback is adjusted to the task requirements (Ribot-Ciscar et al., 2009). Thus, muscle spindle output is a source of afference that is potentially modifiable by training, as shown under active conditions (Dimitriou, 2016). It is pertinent to explore whether this occurs under passive or imposed conditions, especially as this could be useful in rehabilitation. This potential has been demonstrated in a study by Wong et al. (2012), who found that imposed movement training augmented motor learning, even more than under active conditions.

Here, we aimed to investigate, under passive, imposed conditions, whether proprioceptive learning involves a fusimotor-induced change in the coding of position and/or movement from muscle receptors. This would help to determine whether neuroplasticity induced by movement training is limited to central changes or whether the effects of learning also occur peripherally. We hypothesized that passive movement training of the ankle would increase proprioceptive acuity and that proprioceptive afferent signals, recorded in single muscle afferents via peripheral nerve microneurography, would change their firing after the same passive movement training.

## Materials and Methods

Participants gave written informed consent and were paid for their participation. The study was approved by the local ethics committee (Comité de Protection des Personnes Sud-Méditerranée I) and performed in accordance with the Declaration of Helsinki.

### Experimental setup

**Figure 1.**
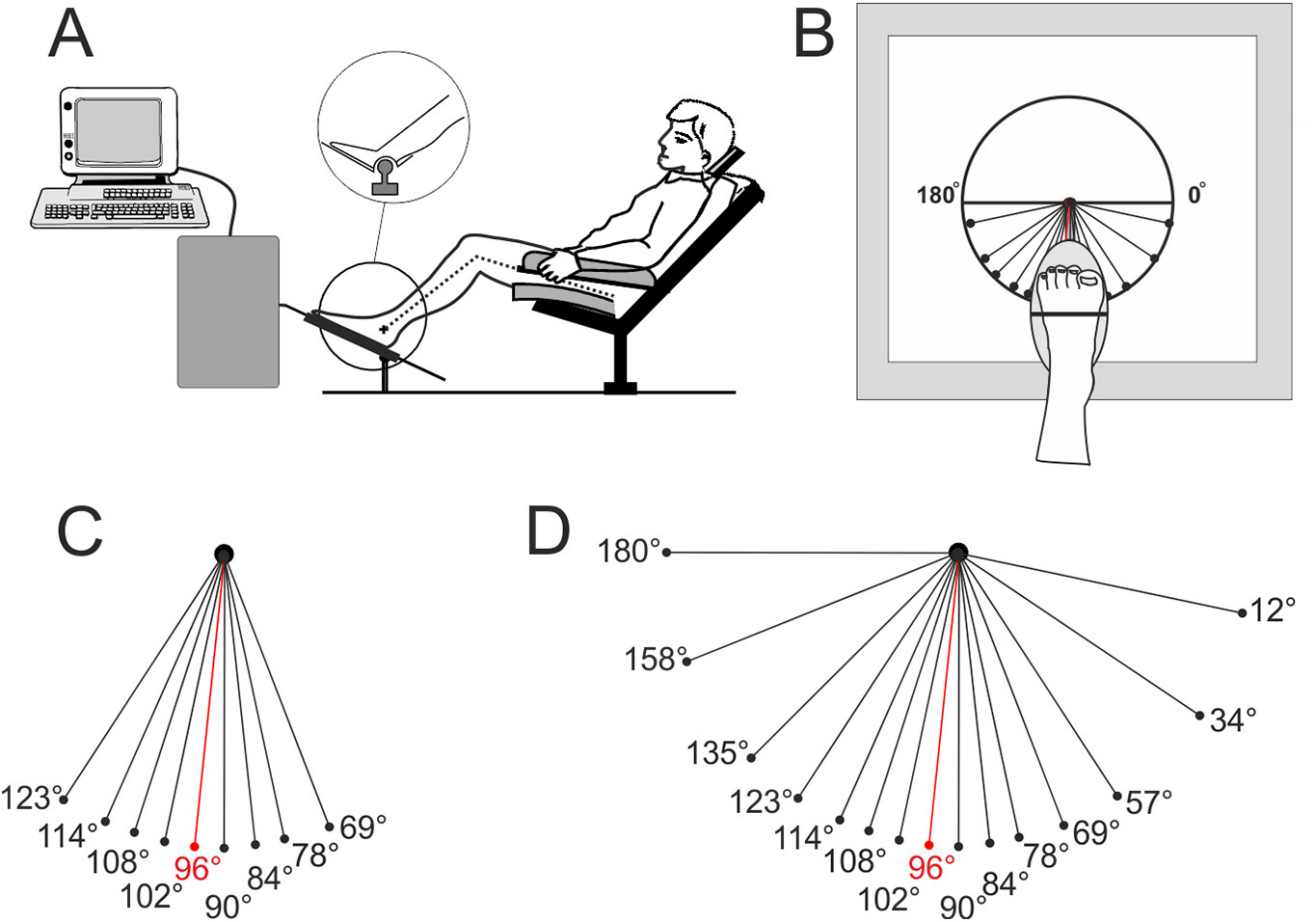
Setup of the experiment. ***A***, Standardized setup for both the psychophysical and microneurography experiments. ***B***, View of the foot in ***A***, with the positions of the movement directions. ***C***, Movement directions used in the psychophysical experiment. ***D***, Movement directions used in the microneurography experiment. Note that the reference movement direction was 96° (***C***, ***D***, red) for both experiments.

Participants were seated in an armchair with their legs positioned in cushioned grooves, so that a standardized position could be maintained without muscle activity (Fig. 1*A*). The knee joint was at an angle of 120–130°. The right foot was laid on a stationary plate, and the left foot was laid on a rotating pedal connected to a servo-controlled robot that permitted the imposition of ramp-and-hold ankle movements in different directions. This foot-movement robot (Rematique) had a metal rod that moved freely and smoothly in a two-dimensional plane and could rotate around its own axis (360°). The lower end of the pedal rested on a ball joint. The metal rod moved in the two-dimensional front plane of the robot, meaning that a downward movement on the front plane of the robot (90°) created a plantar flexion of the ankle joint. A sideward movement to the left (i.e., 180°) resulted in an eversion (a movement of the sole of the foot away from the median plane) and not a translation of the foot (Fig. 1*B*). The center of rotation of the foot was adjusted and lined up with the center of rotation of the ankle joint for each participant.

### Psychophysical experiment

Thirty-two healthy participants (age range, 20–28 years; 20 females) participated in this study. Sixteen participants were randomly assigned to the cued group (mean ± SD age, 22 ± 3 years), and 16 (mean ± SD age, 24  ± 4 years) to the control group. The participant’s left foot was brought to different positions by means of ramp-and-hold plantar flexion movements (amplitude, 5°; velocity, 6°/s; hold phase, 1 s) and then returned to the initial position. The direction of these ankle movements varied so that the attained positions were circularly arranged in the plantar flexion space. The 96° direction brought the foot to the “reference” position, and four other directions on each side of the reference brought the foot to eight different “test” positions (123°, 114°, 108°, 102° and 90°, 84°, 78°, 69°), with a 90° direction corresponding to a pure plantar flexion, from 102° to 123° corresponding to leftward movements, and 84° to 69° to rightward movements (Fig. 1*C*).

The experimental procedure consisted of pairs of movements (trials) where the participant’s foot was first moved to a reference position and held for 1 s, then it was brought back to the initial position. Next, the foot was moved to a test position where the participant made a two-alternative forced choice judgment about the direction [i.e., which side (left or right) the second position was on with respect to the first one] and held for 1 s. The test and reference positions in the pair were presented in random order. Each test was imposed 15 times, so a whole dataset composed a total of 120 pairs of positions.

This experimental procedure was applied before a training session (“pretraining”) and after a training session (“post-training”). To familiarize all participants with the procedure, 10 practice trials were performed at the beginning of the experiment to ascertain that they understood the task. Then, the pretraining session was conducted. Each test/reference pair of positions was separated by 15 s, and after each series of 20 pairs, a 1 min rest period was given. After a short pause, passive movement training was conducted, where each test location was imposed 5 times and the reference position 10 times, in a random order. In the “cued group” (*n* = 16), a sound beep was delivered each time the reference (96°) was given, and the participant was instructed to focus on the actual position and to learn to recognize the reference. In the “control group” (*n* = 16), the same positions were imposed, but no auditory cue was delivered. After another pause, the post-training session was conducted, with the same timings. During the tests, the participants were blindfolded and wore noise-cancelling headphones (Bose) to eliminate any auditory cues. The duration of the entire experiment was ∼1 h and 30 min.

### Analysis of psychophysical data

**Figure 2.**
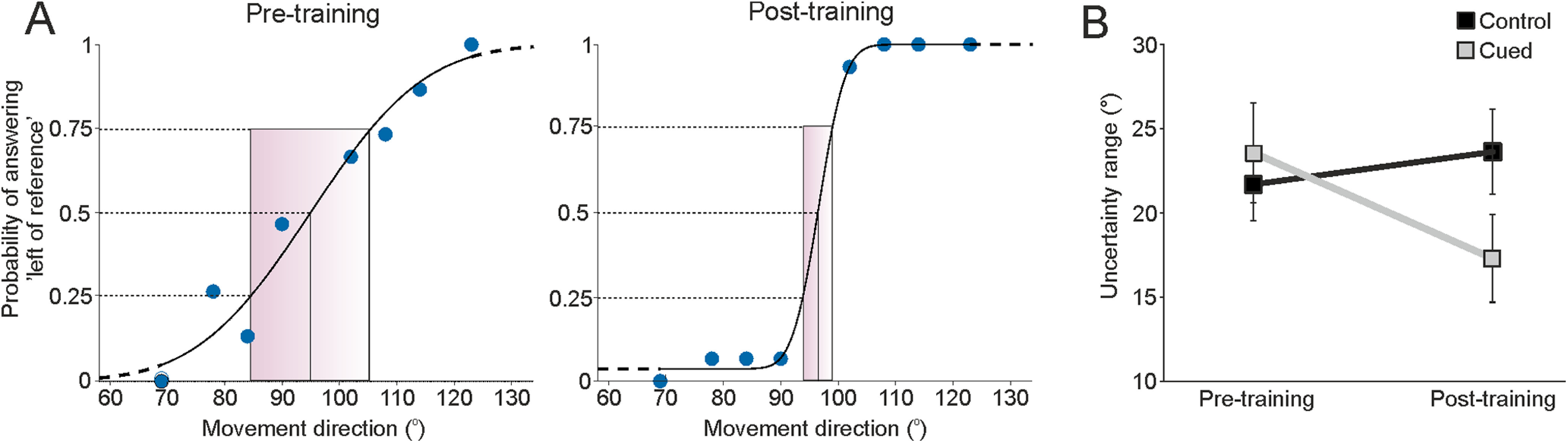
Results from the psychophysical experiment. ***A***, An example of data from a single participant before training (pretraining, left) and after training (post-training, right). The blue dots indicate the answers for each movement direction, and the red box indicates the uncertainty range (between 25% and 75%). ***B***, Group data for the psychophysical experiment, showing both the control (*n* = 16) and cued (*n* = 16) groups and the change in the uncertainty range pretraining and post-training. Error bars show ±SEM.

To evaluate and compare proprioceptive acuity before and after training, a psychometric function was estimated by fitting each participant’s set of responses at each test position to a binomial model using a cumulative normal distribution function, producing a psychometric curve for each participant. To fit the psychometric curves to the data, we used Psignifit toolbox implemented in MATLAB (MathWorks). To quantify proprioceptive acuity, the following two parameters were extracted from each psychometric curve: (1) the point of subjective equality (PSE); and (2) the slope of the curve. (1) The PSE corresponds to the test position perceived by the participant as equal to the reference (i.e., it corresponded to the test position for which the participant gave 50% of their answers as “left”; Fig. 2). (2) The slope was determined by the “sigma” (uncertainty range) that is the difference (in degrees) along the testing *x*-axis between the 25% and 75% probabilities of reporting that the foot position was to the left of the reference position (Figs. 1, positions, 2, range). The slope of the curve is inversely related to a participant’s discrimination sensitivity, where the steeper the slope, the better the participant discriminates the position.

### Microneurography experiment

Single-unit muscle afferent activity was recorded by microneurography (Hagbarth and Vallbo, 1968; Bergenheim et al., 1999). An insulated tungsten microelectrode (impedance, 0.3–1 MΩ tested at 1 kHz; tip diameter, ∼5 μm; length, 30 mm; FHC) was inserted into the common peroneal nerve at the level of the popliteal fossa in nine healthy participants (mean age, 24 years; age range, 20–31 years; four females). Neural activity was recorded with a high gain (100,000×), a bandpass of 300–3000 Hz, and was sampled at 20 kHz. Recordings were made from nine muscle spindle primary (Ia) afferents, as it was only possible to train one participant once, without having consequent training effects; however, two units were rejected, as there were missing data (the full experiment was not completed, before losing the unitary recording).

The classification of Ia muscle afferents was deduced from the response to passive ramp-and-hold (5°, 5°/s) and sinusoidal movements (5°, 5 Hz), during which the afferents exhibited a high dynamic sensitivity and silence during muscle shortening (Edin and Vallbo, 1990b). The absence of any muscle activity was monitored throughout the experiments by recording surface electromyographic (EMG) activity. Two pairs of surface electrodes were placed over the tibialis anterior (TA) and gastrocnemius soleus muscle bellies. The EMGs were recorded with a high gain (5000×) and a bandpass of 3–3000 Hz, and were sampled at 10 kHz. The participant’s electrodermal activity (EDA) was monitored throughout the experiment, using two surface electrodes placed on each side of the left hand (gain, 500×; bandpass, 0.1–100 Hz; sampling frequency, 200 Hz). This measure was used to monitor the state of the participant throughout the experiment, as was habitually done in our microneurography investigations (Ribot-Ciscar and Ackerley, 2021), and it also served as an objective way of measuring how engaged the participant was in the experimental conditions. EDA measures skin sweat gland activity and is thought to be a psychophysiological index of the autonomic nervous system, where changes in emotional and cognitive states can be monitored (Critchley, 2002).

The testing procedure consisted of a series of 15 ramp-and-hold plantar flexion movements, of 5° in amplitude at a 6°/s constant angular velocity and a 1 s hold phase, which were imposed at the ankle joint in a random order and separated by ∼4 s (Fig. 2, examples). As in the psychophysiological experiment, the direction of movements was varied. The same reference (96°) was used, with the following seven other positions on each side of the reference: 180°, 158°, 135°, 123°, 114°, 108°, and 102°, corresponding to leftward directions, compared with reference; and 90°, 84°, 78°, 69°, 57°, 34°, and 12°, corresponding to rightward directions (Fig. 1*D*). The range of positions was larger than in the psychophysiological experiment. This enabled us to obtain cosine movement tuning functions (see below) with high accuracy, whatever the preferred direction of the recorded afferent (i.e., 96° for afferents from the TA muscle and 59° for afferents from the extensor digitorum longus (EDL) muscle, which are the two main muscles targeted when recording the common peroneal nerve (Bergenheim et al., 2000). The extended range of positions was used in all test conditions during the microneurography experiment, to gain directional tuning curves to compare between conditions. The microneurography training session was identical to the training given in the psychophysical experiment and therefore used the slightly reduced set of movements (Fig. 1*D*), meaning that it was not possible to fit a directional tuning curve to these data; thus, the responses captured during the training could not be analyzed in a comparable way to the other conditions.

### Microneurography protocol

Each experiment first began with a “baseline” procedure, where the participant was asked not to pay attention to the movements imposed at the ankle joint, which served as a control resting measure of muscle afferent responses during a fully passive, nonattentive situation. The second procedure was called “direction discrimination,” where the participant was asked to pay attention to the direction of movement and to inform the experimenter after each trial whether they perceived a direction to the left or to the right, compared with vertical. This served as a control passive, imposed movement condition, where the participant had to engage in a novel proprioceptive task. This was followed by a training session (“training,” identical to the psychophysical experiment training) where the ramp movements were imposed two times each and the reference 10 times, all in a random order. During this training, each time the reference (96°) was imposed, a sound beep was delivered, and the participant was instructed to focus on learning the direction of the reference, to recognize it afterward. Finally, the “reference recognition” procedure was run, where the participant had to focus on the movement direction and to say whether it was the trained reference or not. Thus, to successfully complete the reference recognition task, passive proprioceptive learning had to take place.

### Analysis of microneurography data

**Figure 3.**
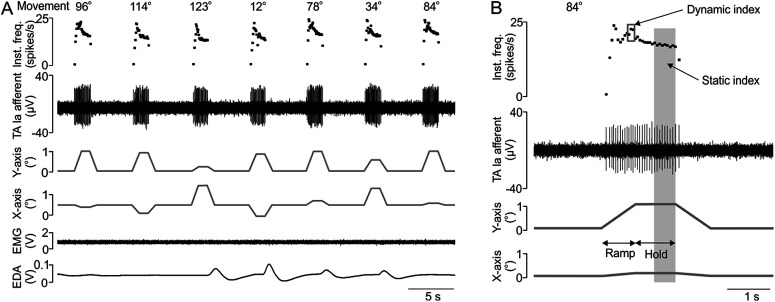
Examples of unitary muscle afferent responses to imposed movements. ***A***, A series of seven imposed movements in real time is shown, with the corresponding TA Ia afferent firing and instantaneous frequency, as well as the *x*-axis and *y*-axis movements of the robot, and EMG TA muscle and EDA (from the hand) responses. ***B***, A zoomed-in example is shown of the last movement in the series in ***A*** to demonstrate the typical response of a Ia muscle afferent with the dynamic index response to the ramp movement and static index response to the hold position.

The data were processed offline using Spike 2 (CED). For each movement of each afferent, the dynamic and static indices were extracted (Fig. 2*B*; as routinely obtained in such analyses: Edin and Vallbo, 1990a; Grill and Hallett, 1995; Kakuda and Nagaoka, 1998). The dynamic index was defined as the peak level of instantaneous firing at the end of the ramp movement, as given by the average of the three highest-frequency points (Fig. 3*B*). The static index was the mean frequency of discharge measured during the last 0.5 s of the hold position for each movement direction (Fig. 3*B*).

As in previous work, both indices were fitted to a directional tuning curve (Fig. 2*C*; Ribot-Ciscar et al., 2003). Here, a cosine curve was fitted to the 15 values of each index by means of a multiple regression analysis applied to find the constants *b*0, *b*1, and *b*2 for the tuning equation: *F* = *b*0 + *b*1 sin θ + *b*2 cos θ, where *F* corresponds to the discharge frequency, *b*0 is the mean of rates found in change in direction to the 15 movement directions, *b*1 is the *y* component of the maximum discharge in the preferred direction, *b*2 is the *x* component of the maximum discharge, and θ is the angle (direction) of the tested movement (Fig. 2*C*). Finally, the amplitudes (maximum – minimum discharge frequency) of both the dynamic and static directional tuning curves were extracted from the fitted curve in each the three conditions (baseline, direction discrimination, reference recognition) per afferent tested.

We also analyzed the EDA data that were obtained concurrently during microneurography. For the test conditions (which all took virtually the same time to conduct), we selected the 120 s of EDA that related to the task duration, conducted baseline correction via removing the DC offset of the signal, and rectified the data. We then measured the area under the curve (in arbitrary units) for each participant over the three test conditions.

### Statistical analysis

We used the same approach to statistically compare both the psychophysical results and microneurography results. First, each set of data were tested for normality. Although the data were relatively normally distributed, a couple of variables did not pass the Shapiro–Wilk normality test (*p* < 0.05). For these, we used Wilcoxon signed-rank test to compare differences in sets of data and Friedman ANOVA for group data. Where data were normally distributed, we used repeated-measures one-way ANOVA tests and linear regression was used to examine trends between variables over the microneurography conditions. Pearson’s tests were used to explore correlations between data. All tests were controlled for multiple comparisons using false discovery rate corrections. For all statistics, 95% confidence intervals (CIs) are given.

## Results

### Psychophysical experiment

In our psychophysical experiment, proprioceptive acuity improved with training only in the cued group. Figure 2*A* shows an example of the psychometric function of proprioceptive acuity in one participant before and after training. In the pretraining condition (Fig. 2*A*, left), the participant perceived the position with good accuracy, as the PSE was 95°, meaning that they perceived the foot to be only slightly shifted to the right of its actual position at the reference (96°). The participant’s ability to discriminate positions was over a range of 31° (uncertainty range). After cued training, the participant’s proprioceptive acuity improved, as demonstrated by an increase in the slope of the psychometric curve and an associated decrease of the uncertainty range to 7° (Fig. 2*A*, right). Further, the participant perceived their foot position as slightly tilted, but this time toward the left of the reference (PSE = 97°). This example was representative of the population of participants of the cued group, where the uncertainty range significantly decreased from 24° (±3 SEM; CI, 17–30) to 17° (±3 SEM; CI, 12–23) after training (Wilcoxon test = −94, *N* = 16, *p* = 0.008; Fig. 2*B*), while the PSE did not change from before (96 ± 2°; CI, 95–97) to after (96 ± 3°; CI, 95–97) training (Wilcoxon test = 2, *N* = 16, *p* = 0.969).

The same discrimination tests were conducted on the control group. The pretraining results obtained with this population of participants were comparable to that of the cued group (Fig. 2*B*, pretraining). The PSE was almost identical at 96 ± 2° (CI, 95–97) and the uncertainty range was slightly inferior (22 ± 2°; CI 17–26) although not significantly different from the cued group (Wilcoxon test = −18, *N* = 16, *p* = 0.889), for the first procedure. Between the two test periods, the control group participants were subjected to the same changes of foot position, but without being cued for learning. For the control group, the proprioceptive acuity of the participants did not change significantly between the two test procedures, where the uncertainty range slightly increased (24 ± 3°; CI, 18–29), but not significantly (Wilcoxon test = 18, *N* = 16, *p* = 0.659; Fig. 2*B*), and the PSE did not change (95 ± 4°; CI 93–97; Wilcoxon test = 16, *N* = 16, *p* = 0.889).

### Microneurography experiment

The responses from seven muscle spindle primary afferents were included (three from EDL muscle and four from TA muscle), which were analyzed through 15 ramp movements of various directions ranging from 12° to 180°, where 90° corresponded to a pure plantar flexion and 180° to an eversion of the foot to the left. Figure 3*A* demonstrates an example of an Ia muscle afferent unitary response from TA muscle over half of the protocol sequence, showing a variety of movements and the corresponding firing of the afferent. We were careful to make sure that there was no concomitant EMG present and where necessary, occasional trials were removed because of EMG activity; however, this was 3 trials out of a total of 315 trials, which did not affect the analyses. The lack of EMG is seen in Figure 3*A*, as well as low electrodermal activity. Figure 3*B* shows an extended time period of one movement of the same unit, where the different parts of the ramp-and-hold response were measured, including the dynamic index, measured at the end of the ramp movement, and the static index, measured at the end of the hold phase. These measures were obtained for each unit over all the movement angles and for each condition.

**Figure 4.**
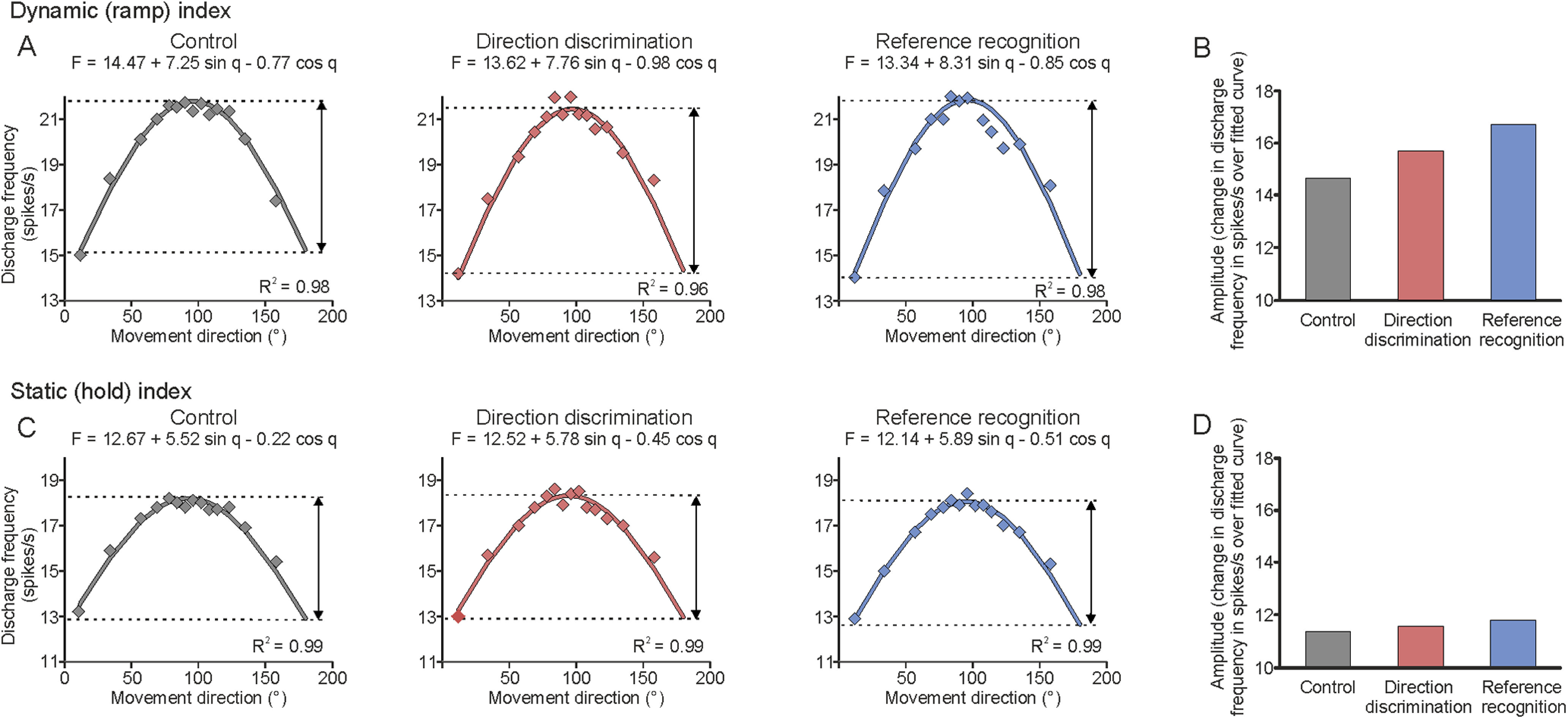
A full example of fitted TA Ia unitary muscle afferent activity to the cosine curves over all the movement directions in both the dynamic and static indices. ***A***, The three curves show the data point responses of the unit to the 15 movement directions and fitted regression curve, for the control, direction discrimination, and reference recognition conditions for the dynamic index. The amplitude of the measured dynamic index for each condition is indicated by the arrow, with the results shown in ***B***. ***B***, The amplitude of the response over each of the conditions for the dynamic index. ***C***, The three curves show the data point responses of the unit to the 15 movement directions and fitted regression curve for the control, direction discrimination, and reference recognition conditions for the static index. The amplitude of the measured static index for each condition is indicated by the arrow, with the results shown in ***D***. ***D***, The amplitude of the response over each of the conditions in the static index.

The dynamic and static indices were analyzed separately, and Figure 4 shows an example from the firing patterns of a different Ia afferent originating in the TA muscle. For each condition, a cosine tuning curve was fit over the movement angles for both the dynamic (ramp) index (Fig. 4, top) and in the static (hold) index (Fig. 4, bottom), where we measured its amplitude: the difference between the minimum and maximum frequencies of each curve. The tuning curves for each unit in each condition were all significant (*R*^2^ between 0.68 and 0.98 for the dynamic index and *R*^2^ between 0.72 and 0.99 for the static index), showing good fits of each muscle spindle response over the movement directions. The example unit in Figure 4 demonstrates that the discharge frequency changed over the movements, which were fit well by the curve, and that this was modulated over the different conditions (control, direction discrimination, reference recognition; Fig. 4, compare *A*, *B*). There was a gradual increase in the amplitude of this response from control to reference recognition in the dynamic index, but this did not occur in the static index (Fig. 4*B*,*D*, respectively).

**Figure 5.**
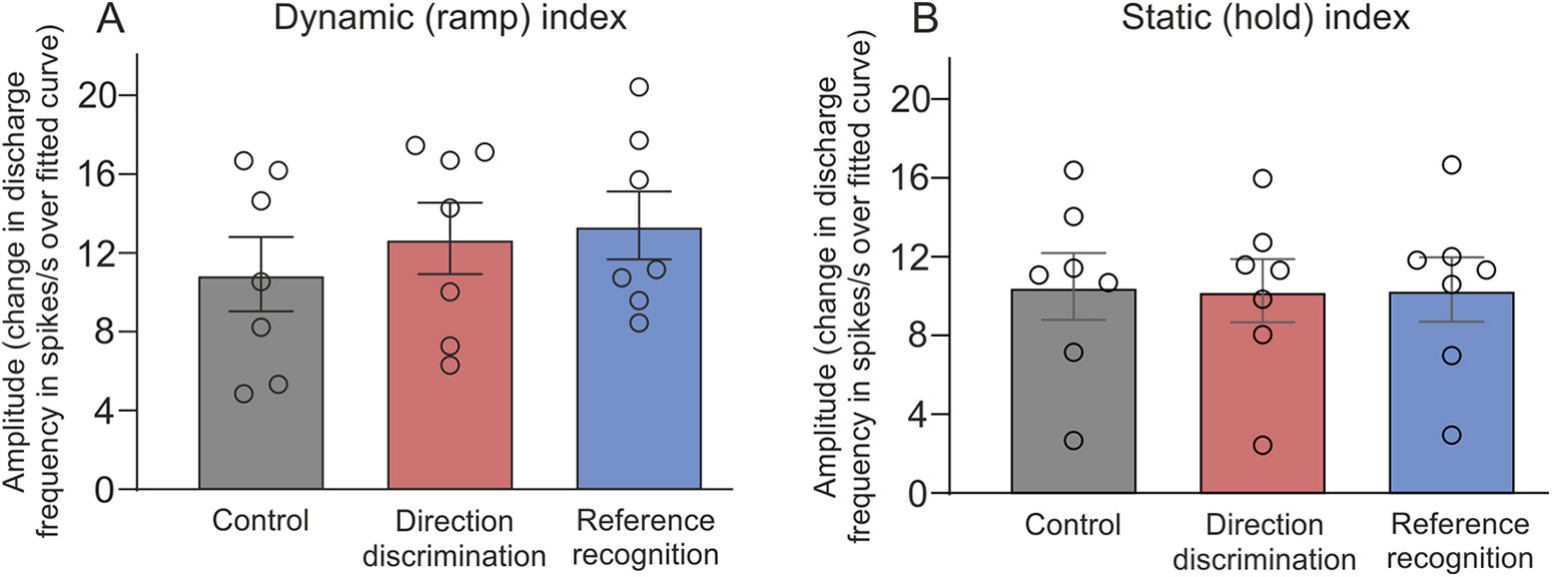
Group responses for Ia muscle afferents during the dynamic and static indices. ***A***, ***B***, The amplitude data (minimum – maximum discharge derived from the fitted cosine curve to all the movements) from seven Ia afferents is shown for the dynamic index (***A***) and static index (***B***). There was a main effect of condition for the dynamic index, but not for the static index. Error bars show ±SEM.

A similar pattern was found in the group data, where there was a significant effect of condition for the dynamic index (Friedman ANOVA: *F*_(3,6)_ = 7.14; *p* = 0.027; control: mean = 10.9; CI, 6.3–15.5; direction discrimination: mean = 12.7; CI 8.3–17.2; reference recognition: mean = 13.4; CI, 9.2–17.6; Fig. 5*A*), corresponding to a significant increase in the amplitude of the response from control to reference recognition (*p* = 0.008; control to direction discrimination, *p* = 0.095). There was no significant effect of condition on the static index (Friedman ANOVA: *F*_(3,6)_ = 1.14; *p* = 0.620; control: mean = 10.5; CI, 6.3–14.6; direction discrimination: mean = 10.3; CI, 6.3–14.2; reference recognition: mean = 11.3; CI, 6.3–14.3), as can be seen in Figure 5*B*. We examined the responses that the participants gave in the direction discrimination and reference recognition sessions. In the direction discrimination condition, where the participant was required to say whether they perceived the foot movement to the left or right of vertical, they made on average 3.0 (SEM, ±0.4) errors of 15 movements. In the reference recognition session, where the participants had to say whether the movement imposed was the reference or not, they made on average 2.8 (SEM, ±0.5) errors of 15 movements.

**Figure 6.**
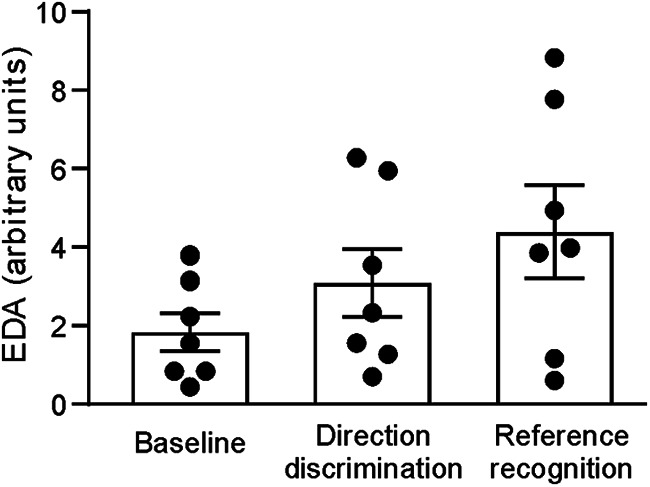
Electrodermal activity during microneurography test conditions. EDA was measured during the collection of microneurography data, where EDA significantly increased over the conditions. Error bars show ±SEM.

We additionally measured EDA throughout the microneurography conditions to obtain measures of sweat gland activity, which is linked to processes like attention and arousal, during each task. Our results show that EDA was low in the baseline condition, where the participant was instructed to remain relaxed. The level of EDA increased over the following direction discrimination and reference recognition tasks (Fig. 6; linear regression: *R* = 0.44, *F*_(1,19)_ = 4.42, *p* = 0.049). There was a significant main effect over the conditions (ANOVA: *F*_(2,12)_ = 4.73; *p* = 0.031; control: mean = 1.8; CI, 0.7–3.0 5; direction discrimination: mean = 3.1; CI, 1.0–5.2; reference recognition: mean = 4.4; CI, 1.5–7.3), where a *post hoc* corrected significant difference was found between the baseline and reference recognition conditions (*p* = 0.010). We explored whether the muscle afferent dynamic index correlated with EDA, but we found no significant effect (Pearson’s *r* = −0.01; CI, −0.44-0.42; *p* = 0.955).

## Discussion

The present study demonstrates that proprioceptive acuity can be improved at the level of the ankle joint by imposed training, which consisted of learning to recognize a passively applied foot movement in a specific direction. We postulate that the observed increase in acuity is related to an increase in muscle spindle sensitivity, as seen in small, yet significant, differences in the firing of muscle spindle afferents.

### Enhanced proprioceptive acuity of the ankle following passive movement training

The acquisition of a new motor skill is associated with changes in the perceptual system, where motor learning drives sensory plasticity (Ostry et al., 2010; Ostry and Gribble, 2016). The reverse is also found, where perceptual learning drives changes in the motor system, for example, in proprioceptive training where a participant’s hand is passively moved to reproduce a trajectory, the rate of motor learning is improved (Wong et al., 2012). A recent study has shown that somatosensory influences can play a major role in the process of learning at an early stage of motor skill acquisition (Bernardi et al., 2015). Bernardi et al. (2015) showed that passive arm movement training improved unseen target-reaching performance and that this training was equivalent to a classical motor learning task, using active movements. The correct direction of movement was learned regardless of whether the movement was active or passive, showing the specific role and importance of precise peripheral afferent sensory information in learning. This principle means that passive, imposed training can be highly useful, for example, supplementing active training and in situations where individuals have difficulty moving, such as in rehabilitation. It is sufficient to manipulate perceptual learning to improve motor performance, as has been observed via a change in proprioceptive acuity (Nougier et al., 1996; Ostry et al., 2010; Wong et al., 2011, 2012; Darainy et al., 2013; Bernardi et al., 2015). This is why we aimed to explore the incoming afferent signal itself, in conjunction with observing the behavioral effects of passive, imposed learning.

The previous observations have been found at the level of the hand/arm; thus, one may ask whether passive movement training can improve motor control of the foot. The upper limb is involved in fine movements of object manipulation and interactions with the environment, while the control of leg/foot position and movement is implicated more in larger movements, such as posture and locomotion; therefore, such differences in function may produce differences in training outcome. Here, we extended the work of Bernardi et al. (2015) by applying passive movement training during ankle joint movement to recognize a particular movement direction marked by an auditory cue. This auditory cue mimicked the reinforcement cue delivered by Bernardi et al. (2015) to signify the proprioceptive target zone. Similar to their work, we found that passive movement training modified proprioceptive acuity at the ankle.

Concerning our paradigm, the way in which we characterized the proprioceptive acuity at the level of the ankle had been previously validated at the level of the arm (Wilson et al., 2010), where participants made repeated judgments about different directions of movements with respect to a proprioceptive reference direction. We performed our psychophysical proprioceptive tests in two groups of participants before and after a period during which they followed a training procedure (cued group) or had only their foot displaced in the same various directions but without being involved in a task (control group). We found that in both groups of participants, the capacity to discriminate different positions of the foot was equivalent in terms of proprioceptive acuity in the pretraining period. However, proprioceptive acuity was significantly improved after passive movement training in the cued group, but proprioceptive acuity remained unchanged in the control group. This difference between the groups allowed us to control for the repetition of the discriminative task, which we show was not on its own sufficient to improve performance. Therefore, we presently show that it is possible to train participants on a proprioceptive task with passive movements imposed at the ankle, as has been previously observed for the hand (Beets et al., 2012; Wong et al., 2012; Bernardi et al., 2015), demonstrating the more general effect of movement training over the body, thus expanding such passive training benefits to the leg/foot region and opening up the potential for optimized bodily rehabilitation.

### Passive movement training alters muscle spindle sensitivity

Studies have shown that the differential processing of proprioceptive inputs, following learning, results in changes in the connectivity in the somatosensory network of the brain (Nasir et al., 2013; Vahdat et al., 2014). However, there is the possibility that the somatosensory contribution to motor learning also involves a modulation of the proprioceptive sensory feedback itself, such as that demonstrated under active conditions (Dimitriou, 2016). Currently, we aimed to investigate whether learning led to changes in the periphery (i.e., in the information feedback from muscle spindles about movement performance under passive, imposed movements). We found that Ia muscle spindle sensitivity changed after passive proprioceptive training, during our reference recognition task, where the dynamic sensitivity significantly increased, while during the direction discrimination task Ia firing did not change significantly.

The present findings are in line with our previous studies showing that recognizing imposed movements modifies the sensitivity of muscle spindles and that the modification is adapted to the task, making the muscles spindles more static or dynamic depending on the necessity to differentiate specific positions or movement velocities, respectively (Hospod et al., 2007; Ribot-Ciscar et al., 2009). Thus, although the participants were required to be attentive and engage in both proprioceptive tasks before and after training (i.e., direction discrimination and reference recognition, respectively), the reference recognition was more challenging, akin to our previous paradigms (Hospod et al., 2007; Ribot-Ciscar et al., 2009). Our EDA results also showed that the baseline sweat level was low and only the reference recognition task gave a significant increase in EDA. This was a similar trend to the microneurography results; however, these two measures were not significantly correlated, showing that they may be related, but reflect different measures. Further, as EDA was not associated with their muscle spindle sensitivity, it suggests that the present effect was not solely because of the changes in attention, but also occurred as a result of training.

Our finding that the more challenging the passive proprioceptive task, the higher the dynamic gamma drive, is similar to the increase in gamma dynamic drive reported during novel motor activities in behaving animals (Prochazka et al., 1985). Therefore, learning by passive displacement provides a template of the expected sensory consequence of a new motor skill, but also a tuning of the sensory information from the periphery through the top-down control of the muscle spindle sensitivity. The finding that muscle spindle responses showed increases in dynamic sensitivity account for a top-down control involving dynamic fusimotor neurons (Hulliger, 1984) that would enable muscle spindles to more rapidly react to encode movement direction after passive movement training. The modulation in the response of the muscle spindles that we observed was relatively small; nevertheless, this is a classic observation in humans, especially in the passive participant (for discussion, see Ribot-Ciscar and Ackerley, 2021), and we relate the subtle observed effects to the resulting increase in acuity in our psychophysical experiment.

Such changes in muscle spindle sensitivity during learning have also been observed following the adaptation to a visuomotor task where the hand was actively moved to control a visual cursor that has a biased displacement (Dimitriou, 2016). This shows that the gamma-induced changes in muscle afferent activities are also at work under active conditions, with the consequence that the afferents retrieve their capacity to encode muscle length in conditions of adaptation (Dimitriou, 2021). It should be added that, in normal daily activities, movements of the hand are frequently performed with visual feedback, and we know that vision impacts muscle proprioceptive information where the strength of muscle afferent information increases when the proprioceptive channel is the only source of movement information (i.e., when visual cues are absent; Ackerley et al., 2019). This is less so for the legs because of anatomic reasons. The possibility to control the sensitivity of muscle spindles in leg muscles, as we observed here, may have an even greater impact on posture and locomotion since visual cues are less available and relied on in normal daily activities. Further, a recent study by Papaioannou and Dimitriou (2021) has demonstrated that it is not just visual information about one’s own movements that can modify muscle spindle sensitivity, but also visual information about the external target location. This has implications in that therapeutic strategies, under both active and passive conditions, where one’s own bodily movements could be used, but strategies could incorporate external targets and objects to aid in rehabilitation.

### Impact of proprioceptive training on rehabilitation

Proprioceptive training presents a great interest for rehabilitative programs. Movement rehabilitation has focused on motor re-education for a long time, yet it is now accepted to include attention to somatosensory information, where somatosensory improvement leads to subsequent decreases in motor deficits (for review, see Aman et al., 2015). The finding that proprioceptive training can be achieved under passive conditions is important. The potential for similar improvements to those found under active conditions means that such training could be applied to patients with large motor deficits, who have difficulty executing self-movements. For example, in highly impaired stroke patients, 4 weeks of passive proprioceptive training has been shown to modify sensorimotor network in the contralesional brain hemisphere liable to promote recovery of motor function (Dechaumont-Palacin et al., 2008).

The majority of studies showing that it is possible to train proprioception in healthy participants have been achieved at the level of the upper limb. In the field of rehabilitation, such training is of interest to improve function, for example, in stroke patients to improve hand function, including active touch and object manipulation, which facilitates daily activities (Carey et al., 1993). We show here that proprioceptive training may also be achieved at the ankle level, opening up similar possibilities for lower limb rehabilitation. Our demonstration of proprioceptive training at the ankle level therefore has implications in the rehabilitation of posture and locomotion in patients with lower body motor deficits, such as in the recovery of locomotion.

To conclude, the present study shows that changes in muscle afferent input from the periphery can contribute to and support central perceptual and motor learning. We show this under passive conditions using ankle movements, through recording direct muscle afferent activity over training sessions and relating this to psychophysical findings. We suggest that learning involving other different passive strategies, such as imagination or observation, may also trigger a specific control of muscle spindle sensitivity, but this remains to be explored.

## Synthesis

Reviewing Editor: Mark Laubach, American University Decisions are customarily a result of the Reviewing Editor and the peer reviewers coming together and discussing their recommendations until a consensus is reached. When revisions are invited, a fact-based synthesis statement explaining their decision and outlining what is needed to prepare a revision will be listed below. The following reviewer(s) agreed to reveal their identity: Vaughan Macefield.

Two experts in the field of your study reviewed your revised manuscript. One is satisfied with the changes that were made. The other would like you to address some remaining issues that they believe will greatly improve the paper. Their comments, from the discussion forum on your paper, were:

"I believe the authors should attempt one more revision of their manuscript. In the current version, they have modified their view on a rather fundamental aspect of their experimental paradigm, (i.e., arousal rather than attention is what is being manipulated). Addressing my concerns will likely benefit the paper.

In my comments to the authors, I outline three ’major’ and several ’minor’ points. The first major comment is essentially a suggestion for a correlation analysis of existing data; positive results will strengthen the author’s claims. The second major point asks for a clarification of the author’s message regarding the proposed cause of increases in spindle sensitivity: is it concurrent arousal levels or previous training? The authors seem to suggest both at different parts of the discussion (but the existing evidence just point to arousal). I suggest adding behavioral (EDA) data from 5 additional participants in order to help disambiguate the situation. Collecting data from 5 more subjects should not take more than a week (microneurography not required). Again, this suggestion is for the authors’ benefit, since they seem to prefer attributing the observed findings to training rather than concurrent arousal levels. The third major point essentially asks for considering the predictions of a major existing hypothesis (“fusimotor set”) that can explain the current results (occam’s razor...).

The minor points ask for a slight modification to existing text for general readability, and a more accurate representation of existing literature.”

Their review is shown below. Please address all points as best you can. This reviewer and I will review your revised manuscript before a final decision will be made on it. Thank you.

In the current version of the manuscript, the authors have revised their view concerning the nature of their experimental paradigm: the different experimental conditions are associated with different levels of arousal/alertness/effort than levels of attention, and additional data is provided to back up this claim. While the proposed effect sizes on spindle dynamic index are rather weak (as acknowledged by the authors themselves), the effects are statistically significant, and therefore potentially of substantial biological consequence. However, I still have some concerns and comments that I would like the authors to consider.

Major:

1. The main results involve a significant difference in spindle dynamic index and EDA (arousal) between the ’control’ and ’reference recognition’ conditions. Is there any evidence of a relationship between individual arousal levels (i.e., individual EDA data points) and spindle sensitivity? That is, it will be good to show that higher EDA points in the ’reference recognition’ are positively correlated with the higher dynamic index points of the same experimental condition.

2. In the Discussion section (lines 414 - 416), the authors state “... we postulate that the change [in Ia spindle sensitivity] was due to the training...”. In the subsequent paragraph of the Discussion, the authors essentially state that it is the increased arousal during the more difficult ’recognition’ (post-training) task that induced the increases spindle sensitivity. In other words, the demonstrated effect on spindles during the post-training task was due to the more aroused state of subjects during this task, rather than due to any training that preceded. As reported at lines 346-347, there was a significant difference in EDA only between the control and post-training conditions. Therefore, the increased dynamic index during post-training could simply be attributed to the increased levels of arousal during the post-training task (see also point 3 below). The authors need to address this seeming discrepancy in argumentation and generally clarify and justify further their message regarding the observed effects on spindle sensitivity (e.g., was there any effect of learning on spindles or not?). One suggestion is that the authors gather further EDA data from additional participants (perhaps aim for ∼12 subjects in total, including existing data), to be collected without performing microneurography. The additional behavioral data can help disambiguate the experimental conditions. Ideally, in support of the learning/training hypothesis, I would expect a significant difference in EDA between the ’control’ and ’discrimination’ condition, between ’control’ and ’recognition’, and no significant difference in EDA levels between the ’discrimination’ and ’recognition’. This will help convince the reader that training during ’discrimination’ is indeed a likely contributor towards the increased dynamic index observed during ’recognition’.

3. At least at some part of the Discussion, the authors seem to claim that it is the increased effort/arousal/alertness (because of increased task difficulty) that led to the increase in spindle stretch sensitivity in the more demanding condition. The authors’ main claim supports Arthur Prochaska’s ’fusimotor set’ hypothesis and findings based on recordings from the freely-moving cat: more challenging/arousing tasks and behaviors are normally associated with higher levels of dynamic fusimotor drive. Highlighting of the above in the Discussion is important in terms of placing the results in the existing literature, and would also help clarify the authors’ message. Note, however, this does not mean that arousal-based increases in spindle sensitivity cannot “contribute to and support perceptual and motor learning” as the authors suggest.

Minor:

Lines 14-15. Abstract. ...”participants then underwent movement training...”. Seems to me that it is more accurate to describe training here and elsewhere in the manuscript as e.g. “movement discrimination training” or similar, especially since the movements were imposed. If word count is an issue in the abstract, I suggest removal of information about the gender of subjects (e.g., “20 females, 12 males”); this seems too much detail at this point. The same recommendation holds for other places in the manuscript e.g., lines 394-395.

Lines 78-80. I strongly recommend that this sentence reads: “... making the muscle receptors adapt to the behavioral context [REFS] or task goal (Pappaioannou and Dimitriou, 2021)”. The 2021 paper demonstrates spindle tuning according to visually determined goals within the same behavior/context (reaching). This is hardly a trivial distinction: it demonstrates more sophistication in the control of human vs. cat spindles i.e., goal-directed tuning vs. state/context-directed tuning, as indicated by Prochazka and others.

Line 89. There was basically no training involved in the Pappaioannou and Dimitriou 2021 study; the subjects simply performed discrete reaching movements with the hand, following a preparatory delay. I suggest you remove the above reference from this sentence. The same recommendation holds for line 409 of the Discussion.

Line 95 (and line 380). I suggest you replace “Presently” with “Here"

Line 104. Perhaps missing a “Participants” paragraph title?

Line 456. (“... the external target movement”). I suggest removing the word “movement” or replacing it with “location” or “position”. The external targets were not moving in the 2021 study.

## Author Response

Synthesis Statement for Author (Required):

Two experts in the field of your study reviewed your revised manuscript. One is satisfied with the changes that were made. The other would like you to address some remaining issues that they believe will greatly improve the paper.

We thank the reviewers and the editor for further consideration of our manuscript. We understand the reviewer’s point of view and we agree with their ideas. We have answered them all and we hope these show the results how we really aimed to write them.

Their comments, from the discussion forum on your paper, were:

"I believe the authors should attempt one more revision of their manuscript. In the current version, they have modified their view on a rather fundamental aspect of their experimental paradigm, (i.e., arousal rather than attention is what is being manipulated). Addressing my concerns will likely benefit the paper.

In my comments to the authors, I outline three ’major’ and several ’minor’ points. The first major comment is essentially a suggestion for a correlation analysis of existing data; positive results will strengthen the author’s claims. The second major point asks for a clarification of the author’s message regarding the proposed cause of increases in spindle sensitivity: is it concurrent arousal levels or previous training? The authors seem to suggest both at different parts of the discussion (but the existing evidence just point to arousal). I suggest adding behavioral (EDA) data from 5 additional participants in order to help disambiguate the situation. Collecting data from 5 more subjects should not take more than a week (microneurography not required). Again, this suggestion is for the authors’ benefit, since they seem to prefer attributing the observed findings to training rather than concurrent arousal levels. The third major point essentially asks for considering the predictions of a major existing hypothesis (“fusimotor set”) that can explain the current results (occam’s razor...).

The minor points ask for a slight modification to existing text for general readability, and a more accurate representation of existing literature.”

Their review is shown below. Please address all points as best you can. This reviewer and I will review your revised manuscript before a final decision will be made on it. Thank you.

Comments from reviewer:

In the current version of the manuscript, the authors have revised their view concerning the nature of their experimental paradigm: the different experimental conditions are associated with different levels of arousal/alertness/effort than levels of attention, and additional data is provided to back up this claim. While the proposed effect sizes on spindle dynamic index are rather weak (as acknowledged by the authors themselves), the effects are statistically significant, and therefore potentially of substantial biological consequence. However, I still have some concerns and comments that I would like the authors to consider.

Regarding our modification from saying ’attention’ to ’arousal’, we were not 100% convinced that this was the best thing to do, as it is difficult to ascertain what exactly is being measured by EDA responses under these situations (Critchley, 2002). However, in light of the reviewer’s thoughts and our further reflection, we would prefer to change the text back to saying ’attention’, as we really feel that this is a better representation of what we varied in the experiment. In general, EDA is believed to measure physiological and/or psychological mechanisms. However, what it actually measures are only changes in skin conductance, via sweat gland activity, which is related to autonomic sympathetic nervous system activity, where the association between psychological state and EDA is more complex and involves further underlying processes. When sweat gland activity increases, it is typically due to a mix of variables and can be attributed to arousal, emotional, cognitive, and attentional changes, but these cannot be fully distinguished (Critchley, 2002).

Thus, we prefer to say ’attention’, as in the previous version and in all our discussions, as this is what we aimed to manipulate. We take on board that this is not a ’pure’ manipulation, but the more difficult task inherently needs more attention. We have modified parts of the Methods and Results to better describe what the EDA data give, including:

Bottom of p. 8/top of p. 9:

’EDA measures skin sweat gland activity and is thought to be a psychophysiological index of the autonomic nervous system, where changes in emotional and cognitive states can be monitored (Critchley, 2002).’

Bottom of p. 14:

’We additionally measured EDA throughout the microneurography conditions, to obtain measures of sweat gland activity, which is linked to processes like attention and arousal, during each task.’

Major:

1. The main results involve a significant difference in spindle dynamic index and EDA (arousal) between the ‘control’ and ‘reference recognition’ conditions. Is there any evidence of a relationship between individual arousal levels (i.e., individual EDA data points) and spindle sensitivity? That is, it will be good to show that higher EDA points in the ‘reference recognition’ are positively correlated with the higher dynamic index points of the same experimental condition.

This is an interesting point about whether there is a relationship between individual EDA and spindle sensitivity - thank you for raising it. We have conducted a number of further analyses to explore this possibility, but we found no correlation whatsoever. We looked at overall correlations and correlations between the specific parts of the experiment, but nothing could be clearly gained from looking at the graphical output and no statistic was significant. Specifically for the correlation between EDA and the dynamic index in the reference recognition condition, we find R2=0.04, p=0.687. We even tried normalizing the data, but still no correlations were found. Although this is interesting to investigate, it does not seem to be sufficient to explain differences in firing, meaning that it was not simply attention that modified the spindle sensitivity, but that the training itself had an effect. There are also differences in whether people are ‘sympathetic nervous system responders’ or not (Donaldio et al., 2012), therefore EDA can be a sensitive measure in some people, but not in others, which does not necessarily have any link with muscle afferent sensitivity. We have added that no correlation was found to the very end of the Results (top of p. 15) and modified the Discussion to reflect this (top of p. 18).

2. In the Discussion section (lines 414 - 416), the authors state “... we postulate that the change [in Ia spindle sensitivity] was due to the training...”. In the subsequent paragraph of the Discussion, the authors essentially state that it is the increased arousal during the more difficult ‘recognition’ (post-training) task that induced the increases spindle sensitivity. In other words, the demonstrated effect on spindles during the post-training task was due to the more aroused state of subjects during this task, rather than due to any training that preceded. As reported at lines 346-347, there was a significant difference in EDA only between the control and post-training conditions. Therefore, the increased dynamic index during post-training could simply be attributed to the increased levels of arousal during the post-training task (see also point 3 below). The authors need to address this seeming discrepancy in argumentation and generally clarify and justify further their message regarding the observed effects on spindle sensitivity (e.g., was there any effect of learning on spindles or not?). One suggestion is that the authors gather further EDA data from additional participants (perhaps aim for ∼12 subjects in total, including existing data), to be collected without performing microneurography. The additional behavioral data can help disambiguate the experimental conditions. Ideally, in support of the learning/training hypothesis, I would expect a significant difference in EDA between the ‘control’ and ‘discrimination’ condition, between ‘control’ and ‘recognition’, and no significant difference in EDA levels between the ’discrimination’ and ‘recognition’. This will help convince the reader that training during ‘discrimination’ is indeed a likely contributor towards the increased dynamic index observed during ’recognition’.

We thank the reviewer for highlighting our lack of clarity before and we understand their concerns. It is evident that our task is complex and it is difficult to attribute the proportion of the spindle sensitivity change to attention and to training. We do have additional EDA data from microneurography experiments that were ‘lost’ part way through, bringing our total up to n = 10 participants. However, on analyzing these data, we find exactly the same pattern, i.e. that there is a significant effect with a post-hoc difference between the control and recognition (post-training). We see the idea behind the reviewer’s proposal that it would be nice to show no significant difference only between the tasks (discrimination/recognition); however, we feel that this process is more complicated. We would predict a general increase in EDA with task difficulty, as shown in our current results, due to increased mental load (Critchley, 2002). The issue is that people show individual reactions to such stimuli and sympathetic activity (and EDA) can vary greatly between individuals, including having ‘responders’ and ‘non-responders’ (Donaldio et al., 2012). This variability, which we see in our present results from 10 participants, gives an overall impression of increases in EDA for the group, with task difficulty, but this is not necessarily true for individuals, where there is great variation, as above.

In our experimental protocol, we included the discrimination condition to modulate how the attentional factor alone likely influenced the descending drive discharge and to be able to vary the effect of training alone in the recognition condition. However, it turns out that it is very difficult to distinguish between the “attention” parameter and the “training” parameter because the two go hand in hand. In the recognition condition, it is more difficult to identify the reference movement, than to say whether the movement is to the right or to the left of the vertical in the discrimination condition, and therefore attention is greater in “recognition” than in “discrimination”. This is shown by the EDA responses and it is known that attentionally demanding tasks evoke increased EDA responses (Critchley, 2002).

We find only a significant difference between baseline and recognition, for both the dynamic index and EDA, but no correlation between these measures. This supports the idea that these processes are different in individuals and that attention is not the only factor here, thus it is reasonable to propose that the training helps explain the firing differences in the recognition situation. Therefore, we maintain the idea that the change in firing was, at least in part, due to training, rather than simply paying attention to the movement and/or attained position. However, we fully take on board that this is a complex situation that cannot be fully disentangled, thus we have revised the discussion at the bottom of p. 17/top of p. 18 to reflect this:

"Thus, although the participants were required to be attentive and engage in both proprioceptive tasks before and after training (i.e. direction discrimination and reference recognition, respectively), the reference recognition was more challenging, akin to our previous paradigms (Hospod et al., 2007; Ribot-Ciscar et al., 2009). Our EDA results also showed that the baseline sweat level was low and only the reference recognition task gave a significant increase in EDA. This was a similar trend to the microneurography results; however, these two measures were not significantly correlated, showing that they may be related, but reflect different measures. Further, as EDA was not associated with their muscle spindle sensitivity, it suggests that the present effect was not solely due to the changes in attention, but occurred additionally as a result of training.”

3. At least at some part of the Discussion, the authors seem to claim that it is the increased effort/arousal/alertness (because of increased task difficulty) that led to the increase in spindle stretch sensitivity in the more demanding condition. The authors’ main claim supports Arthur Prochaska’s ’fusimotor set’ hypothesis and findings based on recordings from the freely-moving cat: more challenging/arousing tasks and behaviors are normally associated with higher levels of dynamic fusimotor drive. Highlighting of the above in the Discussion is important in terms of placing the results in the existing literature, and would also help clarify the authors’ message. Note, however, this does not mean that arousal-based increases in spindle sensitivity cannot “contribute to and support perceptual and motor learning” as the authors suggest.

We fully agree with the reviewer that our observations can be linked to those made in behaving animals by Prochazka et al (1985), particularly that the more challenging our proprioceptive task, the higher the dynamic gamma drive, which is similar to the increase in gamma dynamic drive reported by this work during novel motor activities in cats. We have added the sentence below to the middle of p. 18:

"Our finding that the more challenging the passive proprioceptive task, the higher the dynamic gamma drive, is similar to the increase in gamma dynamic drive reported during novel motor activities in behaving animals (Prochazka et al., 1985).”

Minor:

Lines 14-15. Abstract. ...”participants then underwent movement training...”. Seems to me that it is more accurate to describe training here and elsewhere in the manuscript as e.g. “movement discrimination training” or similar, especially since the movements were imposed. If word count is an issue in the abstract, I suggest removal of information about the gender of subjects (e.g., “20 females, 12 males”); this seems too much detail at this point. The same recommendation holds for other places in the manuscript e.g., lines 394-395.

We agree with the reviewer that it is important to specify that the movements were imposed and therefore we chose to use “passive movement training” and we have added a better description of the training in the abstract. We feel that it is not discrimination training, as the training itself was simply learning a specific movement position.

Lines 78-80. I strongly recommend that this sentence reads: “... making the muscle receptors adapt to the behavioral context [REFS] or task goal (Pappaioannou and Dimitriou, 2021)”. The 2021 paper demonstrates spindle tuning according to visually determined goals within the same behavior/context (reaching). This is hardly a trivial distinction: it demonstrates more sophistication in the control of human vs. cat spindles i.e., goal-directed tuning vs. state/context-directed tuning, as indicated by Prochazka and others.

Thank you for suggesting this clarification, which makes the sentence more accurate. It has been changed.

Line 89. There was basically no training involved in the Pappaioannou and Dimitriou 2021 study; the subjects simply performed discrete reaching movements with the hand, following a preparatory delay. I suggest you remove the above reference from this sentence. The same recommendation holds for line 409 of the Discussion.

We agree that the additional reference is not ideal here and we have removed both instances of it.

Line 95 (and line 380). I suggest you replace “Presently” with “Here"

These have both been modified.

Line 104. Perhaps missing a “Participants” paragraph title?

We have not added ‘Participants’ as a sub-title, as this is a general introduction to the methods and we feel that it would be confusing to have this sub-title here, when the experiment-specific information comes later.

Line 456. (“... the external target movement”). I suggest removing the word “movement” or replacing it with “location” or “position”. The external targets were not moving in the 2021 study.

Thank you for noticing this and we have changed it accordingly to ‘location’.
